# Lympho-vascular invasion in BRCA related breast cancer compared to sporadic controls

**DOI:** 10.1186/1471-2407-10-145

**Published:** 2010-04-16

**Authors:** Marise R Heerma van Voss, Petra van der Groep, Jos Bart, Elsken van der Wall, Paul J van Diest

**Affiliations:** 1 Department of Pathology, University Medical Center Utrecht, The Netherlands; 2 Department of Internal Medicine, University Medical Center Utrecht, The Netherlands; 3 Department of Pathology, University Medical Center Groningen, The Netherlands

## Abstract

**Background:**

Germline mutations in the BRCA1 gene predispose to the development of breast cancer, exhibiting a specific histological phenotype. Identification of possible hallmarks of these tumors is important for selecting patients for genetic screening and provides inside in carcinogenetic pathways.

Since BRCA1-associated breast cancers have pushing borders that prevent them from easily reaching vessels and are often of the medullary (like) type that is known to have a low rate of lympho-vascular invasion (LVI), we hypothesized that absence of LVI could characterize BRCA1 related breast cancer.

**Methods:**

A population of 68 BRCA1 related invasive breast cancers was evaluated for LVI by an experienced breast pathologist blinded to mutation status, and compared to a control group matched for age, grade and tumor type.

**Results:**

LVI was present in 25.0% of BRCA1 related cases, compared to 20.6% of controls (P = 0.54, OR = 1.29, CI 0.58-2.78).

**Conclusion:**

LVI is frequent in BRCA1 germline mutation related breast cancers, but seems to occur as often in sporadic controls matched for age, grade and tumor type. Apparently, these hereditary cancers find their way to the blood and lymph vessels despite their well demarcation and often medullary differentiation.

## Background

About 5-10% of all breast cancer cases are due to a hereditary predisposition. The two most important genes that, when bearing a germline mutation, predispose to breast cancer, are the BRCA1 and BRCA2 genes. It has been estimated that 5.3% of breast cancers occurring in women under forty years and 1.1% of breast cancers in women from 50 to 70 years are due to mutations in either of these genes[1]. Both genes are considered to be tumor suppressor genes that play a role in DNA repair [2-6] and mammary stem cell differentiation[7,8].

Mutation carriers have an increased life-time risk of developing breast cancer of 57% and 40% and of developing ovarian cancer of 40% and 18% for BRCA1 and BRCA2 respectively[9].

BRCA1 related breast carcinomas have a distinct histopathological phenotype. They have been shown to be more often of the ductal and medullary type, of high grade and to show a high mitotic activity index (MAI) and necrosis [10,11]. An expensive growth pattern is also a prominent feature of this phenotype. Pushing margins have been reported to be significantly more often present and to cover a larger area of the tumor in BRCA1 and -2 related breast cancers[12,13]. In addition, specific immunohistochemical, gene expression and genomic alteration profiles have been described putting this hereditary subgroup apart from other breast cancer subtypes. These tumors usually do not express the estrogen and progesterone receptors and are almost always HER-2/*neu *negative ("triple negative")[10]. Furthermore, accumulation of p53[10] and overexpression of vimentin[14], EGFR [15], HIF-1α[16], p-cadherin, and cytokeratins 5/6 and 14[17] are associated with BRCA1 mutations. At the gene-expression level these tumors cluster together with the basal-like subgroup[18]. BRCA2 related breast cancers are most often of luminal type and seem phenotypically harder to recognize[11].

Unraveling genotype, morphology and immunophenotype of BRCA-germline mutation related breast cancer has several advantages. Established biomarkers help the pathologist to recognize these hereditary cancers, which can help to trigger analyzing family history and to decide on mutation testing in patients at borderline risk, based on family history only. Tools that help to select patients for screening are wanted, since genetic screening is time-consuming and expensive. Further, an established phenotype can help to pin down the pathogenicity of so called "unclassified variant" mutations. Lastly, insight into carcinogenetic pathways may offer opportunities to develop new targeted therapies for treatment and prevention of hereditary breast cancer.

Locoregional and systemic spread of breast cancer cells to respectively the lymph nodes and distant organs occurs after invasion of tumor cells into the lymphatic channels and the blood vessels. Although lymphatics and blood vessels of the breast can basically be discerned using immunohistochemical markers, this is in practice not usually done and lymphatic and blood vessel invasion is lumped as "lympho-vascular invasion" (LVI). LVI is present in approximately 15% of invasive ductal breast carcinomas, but its frequency differs widely among studies (5-50%). It is unusual to find LVI in lobular carcinomas[19]. LVI correlates with the presence of lymph node metastases[20,21] and is, not unexpectedly, a poor prognostic sign [22-24]. We hypothesized that LVI is negatively associated with BRCA1 germline mutations for three reasons. First, BRCA1 associated breast cancers are known to have pushing borders. Such an expansive growth pattern would mean that adjacent structures are pushed aside, rather than infiltrated, so that blood and lymph vessels may not be easily reached and invaded by tumor cells. In ER-negative breast cancer it has been shown that the presence of pushing margins correlates with the absence of LVI and lymph node negative status[25]. In endometrial carcinomas LVI has also been associated with a diffusely infiltrative and almost never with an expansive growth pattern[26].

Second, since the presence of LVI strongly correlates with lymph node metastases, low rates of LVI are likely to result in relatively frequent negative axillary nodal status. Indeed, a trend has been reported toward a higher percentage of lymph node negative, BRCA1-positive breast cancers as compared with controls[27]. In addition, the strong correlation between tumor size and nodal status described for sporadic breast cancer patients, was absent in BRCA1 mutation carriers[28].

Third, BRCA1 related breast cancers are frequently (11-19%) of the medullary tumor type, in contrast to 1% of sporadic cancers[29]. A high percentage of BRCA1 mutations has indeed been reported in medullary breast carcinomas[30]. Medullary carcinomas are associated with a significantly lower LVI rate of 6%[31].

Five studies have previously evaluated LVI in BRCA related breast cancer, some finding more and some less LVI compared to controls, but significance was not reached [13,32-35], due to small populations (n = 8-32) and lack of correction for confounders like grade, thus not allowing definite conclusions.

We therefore set out to compare frequency of LVI in a large set of BRCA1 related breast cancers with well matched sporadic controls.

## Methods

### Study group

A population of 68 patients with hereditary invasive breast cancer, due to a germline mutation in the BRCA1 gene, was studied. A control group of 68 breast cancer patients unselected for family history and with no known mutation (further denoted "sporadic") was selected by case matching for age, tumor type and histologic grade as much as possible. All tumors could be coupled to a tumor with the same grade. The next step was matching for age, using a window of 5 years. Some tumor types were so rare, that after matching for age, no match for type could be found. They were therefore matched to invasive ductal carcinomas. Matching for these features was deemed necessary because these characteristics are significantly different between sporadic and BRCA related tumors and have been shown to be associated with the presence of LVI[36]. We excluded all "sporadic" cases for which a strong family history of breast cancer in the pathology report or electronic patient files was mentioned and all cases of which cumulative breast cancer risk exceeded 30% based on family history in the patient file[37]. MAI was assessed as before[38]. Growth pattern was reported to be expansive if pushing margins were observed in >50% of the tumor circumference[13]. Anonymous use of redundant tissue for research purposes is part of the standard treatment agreement with patients in our hospitals[39]. The protocol for use of the redundant tissue was approved by the science committee of the UMC Utrecht Biobank. Clinical data were retrieved from the pathology report and patient files. 71.3% of patients underwent an axillary lymph node dissection, the remaining 28.7% only underwent a sentinel node biopsy Breast conserving therapy was performed in similar rates in hereditary and sporadic patients.

### Lympho-vascular invasion

All available Hematoxylin-eosin (H&E) stained slides of all tumors were retrieved from our archive and screened for LVI by an experienced breast pathologist (PvD) blinded to BRCA mutation status. For the determination of LVI status we used criteria for identification as postulated by Rosen: "Evaluation is limited to tissue peripheral to the carcinoma, intralymphatic tumor emboli usually do not conform exactly to the space in which they lie, endothelial nuclei should be present and coexistent blood vessels are confirmatory evidence."[40]

Since many of the hereditary cases and some of the sporadic cases were referrals for which only one slide with tumor was available, we selected for each matched pair (case and control) the same number of slides to end up with a comparable number of slides assessed for LVI in both groups. For all patients with only one slide available, we selected for the match the H&E from the block on which immunohistochemistry (IHC) had been done. If no IHC had been done, the slide with the largest tumor area was selected. Although not including all slides might result in some false negatives and thereby lead to an underestimation of LVI frequency in our study group, this would apply to both the hereditary and sporadic in a similar manner and thereby does not influence our comparison between both groups.

### Statistics

Frequencies of LVI in hereditary cases and sporadic controls were compared by Chi-square test and Fischer exact test and odds ratios (OR) were calculated with 95% confidence intervals. Continuous data for both groups (e.g. MAI) were compared by Mann-Whitney U test. When features were significantly associated with both BRCA mutation status and the presence of LVI, statistical analysis of these possible confounders took place by calculating ORs stratified for specific subgroups (corrected by Mantel-Haenszel procedure) and logistic regression for significant features. All statistical analyses were performed using SPSS 15.0.

## Results

### Patients and tissues

Anonymous use of redundant tissue for research purposes is part of the standard treatment agreement with patients in our hospital [41], in compliancy with the Helsinki Declaration. The research protocol for this study was approved by the Scientific Advisory Committee of the UMC Utrecht Biobank.

In the BRCA1 group, median tumor size was 1.8 cm compared to 2.6 for controls (p = 0.003), median MAI was 22 vs 20 in controls (n.s.), and median age was 40 in both cases and controls (n.s.). Invasive ductal carcinomas were slightly more prevalent in the sporadic group (n.s.). Table 1 shows the further features of the BRCA1 population and the sporadic control group. BRCA1 related patients more frequently had negative lymph nodes (67.2% compared to 50.8% in controls), but this difference was not statistically significant (P = 0.06). Expansive growth pattern was present in 47% of BRCA1 related tumors, compared to 41% in sporadic controls (n.s.). Multifocality (more than one invasive intramammary tumor nodule) was present in 21.8% of sporadic tumors, compared to 25% of BRCA1 related tumors (n.s.). All BRCA1 related carcinomas had a negative HER-2/*neu *receptor status when assessed, whereas in the control group 23.5% was positive (P = 0.005). Both ER and PR receptor status were more often negative in the BRCA1 related group (72.7% and 75.4% for ER and PR respectively, compared to 48.3% and 54.5% in controls; P = 0.005 and P = 0.016, respectively).

**Table 1:** Characteristics of BRCA1 related breast cancers and sporadic controls evaluated for frequency of lympho-vascular invasion

		Sporadic control group (n = 68)		BRCA1 group (n = 68)				
		**Number**	**Percentage**	**Number**	**Percentage**	**P-value**	**OR**	**95% CI**

Tumour Type	invasive ductal carcinoma	58	85.3%	55	80.9%	0.83		
	invasive (ducto)lobular carcinoma	3	4.4%	3	4.4%			
	invasive medullary and metaplastic carcinoma	7	10.3%	10	14.7%			

Histologic grade	1	3	4.4%	2	2.9%	0.88^a^		
	2	13	19.1%	14	20.6%			
	3	52	76.5%	52	76.5%			

Tumour size	<2 cm	24	35.3%	37	59.4%	0.02^a^		
	2-5 cm	36	52.9%	22	35.9%			
	>5 cm	8	11.8%	9	4.7%			
	unknown	0		4				

Lymph node status	N0	33	50.8%	39	67.2%	0.06^a^		
	N1	21	32.3%	17	29.3%			
	N2	9	13.8%	1	1.7%			
	N3	2	3.1%	1	1.7%			
	unknown	3		10				

Growth pattern	infiltrative	40	58.8%	36	52.9%	0.49	1.31	0.64-2.50
	expansive	28	41.2%	32	47.1%			

HER-2/*neu *status	negative	26	76.5%	31	100.0%	0.005^b^		
	positive	8	23.5%	0	0.0%			
	unknown	34		37				

ER status	negative	28	48.3%	48	72.7%	0.005^a^	0.38	0.18-0.79
	positive	30	51.7%	18	27.3%			
	unknown	10		2				

PR status	negative	30	54.5%	49	75.4%	0.016^a^	0.42	0.19-0.92
	positive	25	45.5%	16	24.6%			
	unknown	13		3				

Lympho-vascular invasion	negative	54	79.4%	51	75.0%	0.54^a^	1.29	0.58-2.87
	positive	14	20.6%	17	25.0%			

a. P-value obtained with a Chi-square test. b. P-value obtained with a Fischer's exact test

### Lympho-vascular invasion

Frequencies of LVI in the BRCA1 related breast cancer and sporadic controls are shown in table 1. 25.0% of hereditary cases were positive for LVI, compared to 20.6% of controls, which was not statistically significant (P = 0.54, OR = 1.29, 95% CI = 0.58-2.78). Also when stratifying for tumor type, we did not find a significant difference between sporadic and BRCA related carcinomas (table 2). There was a much lower rate of LVI in medullary and metaplastic carcinomas.

**Table 2:** Frequency of lympho-vascular invasion (LVI) in BRCA1 associated breast cancer compared to matched sporadic controls, stratified for tumor type.

		Sporadic control group (n = 68)		BRCA1 group (n = 68)		Total (n = 136)				
**Tumour type**	**LVI**	**Number**	**Percentage**	**Number**	**Percentage**	**Number**	**Percentage**	**OR**	**95% CI**	**P-value**

Invasive ductal carcinoma	negative	45	77.6%	39	70.9%	84	74.3%	1.42	0.61-3.32	0.42^a^
	positive	13	22.4%	16	29.1%	29	25.7%			

Invasive lobular carcinoma	negative	2	66.7%	3	100.0%	5	83.3%	0.40	0.14-1.17	1^b^
	positive	1	33.3%	0	0.0%	1	16.7%			

Invasive medullary and metaplastic carcinoma	negative	7	100.0%	9	90.0%	16	94.1%	0.56	0.37-0.87	1^b^
	positive	0	0.0%	1	10.0%	1	5.9%			

a. Chi-square test. b. Fischer's exact test

No significant confounders were identified by stratified analysis and ORs, adjusted for confounding by Mantel Haenszel procedure, were not significantly different from the crude ORs. Therefore, further analysis of confounders by means of logistic regression was deemed unnecessary.

## Discussion

The aim of this study was to evaluate the absence of LVI as a putative biomarker in BRCA1 germline mutation related breast cancer. Our data did however not show the expected lower rate of LVI in BRCA related tumors.

Five studies have evaluated LVI in BRCA related breast cancer. None of the studies found a significant correlation between mutation status and LVI. Only one study statistically corrected for histologic tumor grade, by means of stratification[32]. This study compared 32 BRCA1 related tumors to 334 unmatched controls. LVI was reported in 31.6% and 25.3% of the cases for BRCA1 related and sporadic tumors, respectively. This result was not significant (P = 0.29). Correction for other possible confounders (e.g. age, tumor type and size) did not take place. In four other studies no matching or statistical correction for confounding was performed at all[13,33-35]. Relatively small populations evaluated (n = 8-32), in addition to the lack of correction for grade and other confounders make it impossible to draw definite conclusions from these five studies.

Rates of LVI in the present study (22.8%) were above the average rate described in literature (15%)[19]. We had indeed expected to find a higher rate of LVI than described in literature, since our population consisted mostly of high-grade carcinomas and high grade has been associated with higher rates of LVI. In populations consisting of solely grade 3 breast cancers, 35-46% of patients were reported to contain LVI[22,42-44]. In three of these four studies immunohistochemical staining for endothelial walls was used for identification of LVI, which might explain the higher rates found in these specific studies compared to our study employing just H&E[22,43,44]. Further, there is no real consensus on the definition of LVI, sometimes including intratumoral LVI, interobserver discordance has been reported [45-47] and case selection differed between studies, possibly contributing to the wide range of frequencies reported. Retraction artefacts and intraductal carcinoma are in some cases hard to distinguish from LVI and can lead to false positives whereas tumor emboli filling up the entire lumen of a vessel sometimes make it hard to detect LVI and may result in false negatives. However, topographic conditions, like other accompanying vessels usually help making the diagnosis LVI[45]. In addition, when LVI seems questionable at one location very often there is clearer LVI present elsewhere in the section.

To deal with interobserver discordance as mentioned, the same observer evaluated all slides. Therefore, potential interobserver bias is of little influence on our comparison between sporadic and hereditary breast cancer. In addition, several studies have shown that regarding equivocal cases as negative did not influence the predictive value of LVI[45,46].

An explanation for the fact that we found lower LVI frequencies, than described in the literature for grade 3 cancers, may lie in that fact that we did not evaluate all slides of all tumors, in order to match for number of evaluated slides per case, so that we may have missed LVI in cases where it is not present in all the slides. To get an impression of the magnitude of this potential reduction, we also assessed LVI in all slides available in the sporadic group. We then found an about 1.5 times higher rate of LVI (30.9%) if we considered all available slides in the sporadic group instead of only those slides used after slide number matching.

In order to find a LVI effect contributable to BRCA1 mutation and not to another feature associated with the BRCA1 phenotype, it was deemed necessary to match for features associated with LVI. Unfortunately, this matching has a downside. By case-matching to select controls, we likely preferentially select some hidden BRCA1/2 mutation carriers (age, histologic type and grade effect), as well as some tumors that have no known BRCA mutation but show a similar phenotype (type, grade effect) by e.g. promoter hypermethylation of BRCA1/2 or a mutation in another gene involved in the BRCA pathway. 9-22% of sporadic breast cancers have been described to show promoter hypermethylation of BRCA1 or loss of heterozygosity at the BRCA1 locus and these tumors are largely ER and PR negative, of ductal and medullary type, and high grade [48-52]. These are all also features of tumors with a mutation in BRCA1. One study selected 7 sporadic tumors with a BRCA1 phenotype, and found that 3 out of 7 showed promoter hypermethylation of BRCA1[53]. Selecting "sporadic" controls with a BRCA1 like phenotype might make it harder to find differences between sporadic and BRCA related carcinomas. This might explain why we did not find the expected correlation between expansive growth pattern and BRCA1 mutation status as was described in literature. The absence of a higher frequency of pushing margins in our BRCA1 related group, is a possible explanation for the unexpected similar rates of LVI found in both groups.

We further looked into features that were associated with both LVI and BRCA mutation status such as age, tumor type, tumor size, nodal status, grade, ER and PR [19,21,22,42-44], to exclude and correct for possible confounders. No significant differences were found between cases and controls for age, nodal status and grade. Contradictory with literature, we did not find a significantly higher MAI in our hereditary group. Since cases were matched for tumor grade and MAI (as a constituent of grade) strongly correlates with grade, a difference was not expected here. Invasive ductal carcinomas were slightly more prevalent in the sporadic group. This is due to the fact that case matching could not always be performed for type in the case of rare tumor types and these were matched to ductal carcinomas. Stratification for tumor type shows a much lower rate of LVI in medullary and metaplastic carcinomas, which is consistent with literature[31]. A significantly lower rate of ER, PR and HER-2/*neu *expression was found in the BRCA1 related group. This is consistent with what we know from literature about BRCA1 related tumors, mostly showing a triple-negative receptor status[54]. ER and PR negative status have been associated with high LVI rates. No significant correlation between HER-2/*neu *status and LVI has been described [19,55]. In our study ER status was not significantly associated with LVI and neither was PR or HER-2/*neu *status. When stratified separately for ER, PR and HER-2/*neu *status, no significant differences for LVI status between cases and controls were found.

In our control group tumor size was slightly larger than in the BRCA1 related group. This has not been reported in literature. Since several studies noted that tumor size is significantly associated with LVI, this is a possible confounder. These studies reported high frequencies (58-69%) of LVI in tumors with a diameter bigger than 5 cm[42,43], although the one reporting the highest rates used immunohistochemistry to detect LVI. In our study LVI in tumors bigger than 5 cm was not significantly higher, so this does not likely play an important role here. The significant difference in tumor size between cases and controls was largely based on a difference in distribution between the group up to 2 cm and the group from 2-5 cm, but between these groups no significant differences in LVI rates were found.

## Conclusions

LVI seems to occur as much in BRCA1 germline mutation related breast cancers as in sporadic controls. Apparently, these hereditary cancers find their way to the blood and lymph vessels despite their well demarcation and often medullary type differentiation.

## Competing interests

The authors declare that they have no competing interests.

## Authors' contributions

MRHV collected samples, performed LVI assessment, carried out statistics and data interpretation, and drafted the manuscript. PG participated in the conception and design of the study, supervised collection of samples, and critically revised the manuscript. JB contributed with samples, participated in data interpretation and critically revised the manuscript. EW selected patients, participated in the conception and design of the study, and critically revised the manuscript. PJD performed LVI assessment, participated in the conception and design of the study, supervised statistics, and helped to draft the manuscript. All authors read and approved the final manuscript.

## Pre-publication history

The pre-publication history for this paper can be accessed here:

http://www.biomedcentral.com/1471-2407/10/145/prepub

## References

[B1] Ford D Easton DF Peto J Estimates of the gene frequency of BRCA1 and its contribution to breast and ovarian cancer incidence Am J Hum Genet 1995 57 1457 1462 8533776 PMC1801430

[B2] Scully R Livingston DM In search of the tumour-suppressor functions of BRCA1 and BRCA2 Nature 2000 408 429 432 10.1038/35044000 11100717 PMC2981135

[B3] Thorslund T West SC BRCA2: a universal recombinase regulator Oncogene 2007 26 7720 7730 10.1038/sj.onc.1210870 18066084

[B4] Reliene R Bishop AJ Schiestl RH Involvement of homologous recombination in carcinogenesis Adv Genet 2007 58 67 87 full_text 17452246 10.1016/S0065-2660(06)58003-4

[B5] Wang Y Cortez D Yazdi P Neff N Elledge SJ Qin J BASC, a super complex of BRCA1-associated proteins involved in the recognition and repair of aberrant DNA structures Genes Dev 2000 14 927 939 10783165 PMC316544

[B6] Nagaraju G Scully R Minding the gap: the underground functions of BRCA1 and BRCA2 at stalled replication forks DNA Repair (Amst) 2007 6 1018 1031 10.1016/j.dnarep.2007.02.020 17379580 PMC2989184

[B7] Lim E Vaillant F Wu D Forrest NC Pal B Hart AH Aberrant luminal progenitors as the candidate target population for basal tumor development in BRCA1 mutation carriers Nat Med 2009 15 907 913 10.1038/nm.2000 19648928

[B8] Liu S Ginestier C Charafe-Jauffret E Foco H Kleer CG Merajver SD BRCA1 regulates human mammary stem/progenitor cell fate Proc Natl Acad Sci USA 2008 105 1680 1685 10.1073/pnas.0711613105 18230721 PMC2234204

[B9] Chen S Parmigiani G Meta-analysis of BRCA1 and BRCA2 penetrance J Clin Oncol 2007 25 1329 1333 10.1200/JCO.2006.09.1066 17416853 PMC2267287

[B10] Honrado E Benitez J Palacios J Histopathology of BRCA1- and BRCA2-associated breast cancer Crit Rev Oncol Hematol 2006 59 27 39 10.1016/j.critrevonc.2006.01.006 16530420

[B11] Bane AL Beck JC Bleiweiss I Buys SS Catalano E Daly MB BRCA2 mutation-associated breast cancers exhibit a distinguishing phenotype based on morphology and molecular profiles from tissue microarrays Am J Surg Pathol 2007 31 121 128 10.1097/01.pas.0000213351.49767.0f 17197928

[B12] Lakhani SR Jacquemier J Sloane JP Gusterson BA Anderson TJ van d V Multifactorial analysis of differences between sporadic breast cancers and cancers involving BRCA1 and BRCA2 mutations J Natl Cancer Inst 1998 90 1138 1145 10.1093/jnci/90.15.1138 9701363

[B13] Armes JE Egan AJ Southey MC Dite GS McCredie MR Giles GG The histologic phenotypes of breast carcinoma occurring before age 40 years in women with and without BRCA1 or BRCA2 germline mutations: a population-based study Cancer 1998 83 2335 2345 10.1002/(SICI)1097-0142(19981201)83:11<2335::AID-CNCR13>3.0.CO;2-N 9840533

[B14] Rodriguez-Pinilla SM Sarrio D Honrado E Moreno-Bueno G Hardisson D Calero F Vimentin and laminin expression is associated with basal-like phenotype in both sporadic and BRCA1-associated breast carcinomas J Clin Pathol 2007 60 1006 1012 10.1136/jcp.2006.042143 17105822 PMC1972443

[B15] van Diest PJ Groep P van der Wall E van der EGFR expression predicts BRCA1 status in patients with breast cancer Clin Cancer Res 2006 12 670 10.1158/1078-0432.CCR-05-2098 16428516

[B16] Groep P van der Bouter A Menko FH van der WE van Diest PJ High frequency of HIF-1alpha overexpression in BRCA1 related breast cancer Breast Cancer Res Treat 2008 111 475 480 10.1007/s10549-007-9817-z 18030615

[B17] Arnes JB Brunet JS Stefansson I Begin LR Wong N Chappuis PO Placental cadherin and the basal epithelial phenotype of BRCA1-related breast cancer Clin Cancer Res 2005 11 4003 4011 10.1158/1078-0432.CCR-04-2064 15930334

[B18] Sorlie T Tibshirani R Parker J Hastie T Marron JS Nobel A Repeated observation of breast tumor subtypes in independent gene expression data sets Proc Natl Acad Sci USA 2003 100 8418 8423 10.1073/pnas.0932692100 12829800 PMC166244

[B19] Hoda SA Hoda RS Merlin S Shamonki J Rivera M Issues relating to lymphovascular invasion in breast carcinoma Adv Anat Pathol 2006 13 308 315 10.1097/01.pap.0000213048.69564.26 17075296

[B20] Weiser MR Montgomery LL Tan LK Susnik B Leung DY Borgen PI Lymphovascular invasion enhances the prediction of non-sentinel node metastases in breast cancer patients with positive sentinel nodes Ann Surg Oncol 2001 8 145 149 10.1007/s10434-001-0145-y 11258779

[B21] Gajdos C Tartter PI Bleiweiss IJ Lymphatic invasion, tumor size, and age are independent predictors of axillary lymph node metastases in women with T1 breast cancers Ann Surg 1999 230 692 696 10.1097/00000658-199911000-00012 10561094 PMC1420924

[B22] Schoppmann SF Bayer G Aumayr K Taucher S Geleff S Rudas M Prognostic value of lymphangiogenesis and lymphovascular invasion in invasive breast cancer Ann Surg 2004 240 306 312 10.1097/01.sla.0000133355.48672.22 15273556 PMC1356408

[B23] Woo CS Silberman H Nakamura SK Ye W Sposto R Colburn W Lymph node status combined with lymphovascular invasion creates a more powerful tool for predicting outcome in patients with invasive breast cancer Am J Surg 2002 184 337 340 10.1016/S0002-9610(02)00950-9 12383896

[B24] Fisher ER Anderson S Tan-Chiu E Fisher B Eaton L Wolmark N Fifteen-year prognostic discriminants for invasive breast carcinoma: National Surgical Adjuvant Breast and Bowel Project Protocol-06 Cancer 2001 91 1679 1687 10.1002/1097-0142(20010415)91:8+<1679::AID-CNCR1183>3.0.CO;2-8 11309768

[B25] Putti TC El-Rehim DM Rakha EA Paish CE Lee AH Pinder SE Estrogen receptor-negative breast carcinomas: a review of morphology and immunophenotypical analysis Mod Pathol 2005 18 26 35 10.1038/modpathol.3800255 15332092

[B26] Mannelqvist M Stefansson I Salvesen HB Akslen LA Importance of tumour cell invasion in blood and lymphatic vasculature among patients with endometrial carcinoma Histopathology 2009 54 174 183 10.1111/j.1365-2559.2008.03201.x 19207942

[B27] Chappuis PO Nethercot V Foulkes WD Clinico-pathological characteristics of BRCA1- and BRCA2-related breast cancer Semin Surg Oncol 2000 18 287 295 10.1002/(SICI)1098-2388(200006)18:4<287::AID-SSU3>3.0.CO;2-5 10805950

[B28] Foulkes WD Metcalfe K Hanna W Lynch HT Ghadirian P Tung N Disruption of the expected positive correlation between breast tumor size and lymph node status in BRCA1-related breast carcinoma Cancer 2003 98 1569 1577 10.1002/cncr.11688 14534871

[B29] Eisinger F Jacquemier J Charpin C Stoppa-Lyonnet D Bressac-de PB Peyrat JP Mutations at BRCA1: the medullary breast carcinoma revisited Cancer Res 1998 58 1588 1592 9563465

[B30] Lakhani SR Gusterson BA Jacquemier J Sloane JP Anderson TJ van d V The pathology of familial breast cancer: histological features of cancers in families not attributable to mutations in BRCA1 or BRCA2 Clin Cancer Res 2000 6 782 789 10741697

[B31] Bertucci F Finetti P Cervera N Charafe-Jauffret E Mamessier E Adelaide J Gene expression profiling shows medullary breast cancer is a subgroup of basal breast cancers Cancer Res 2006 66 4636 4644 10.1158/0008-5472.CAN-06-0031 16651414

[B32] Quenneville LA Phillips KA Ozcelik H Parkes RK Knight JA Goodwin PJ HER-2/neu status and tumor morphology of invasive breast carcinomas in Ashkenazi women with known BRCA1 mutation status in the Ontario Familial Breast Cancer Registry Cancer 2002 95 2068 2075 10.1002/cncr.10949 12412159

[B33] Eisinger F Nogues C Guinebretiere JM Peyrat JP Bardou VJ Noguchi T Novel indications for BRCA1 screening using individual clinical and morphological features Int J Cancer 1999 84 263 267 10.1002/(SICI)1097-0215(19990621)84:3<263::AID-IJC11>3.0.CO;2-G 10371344

[B34] de Bock GH Tollenaar RA Papelard H Cornelisse CJ Devilee P van d V Clinical and pathological features of BRCA1 associated carcinomas in a hospital-based sample of Dutch breast cancer patients Br J Cancer 2001 85 1347 1350 10.1054/bjoc.2001.2103 11720473 PMC2375234

[B35] Robson M Rajan P Rosen PP Gilewski T Hirschaut Y Pressman P BRCA-associated breast cancer: absence of a characteristic immunophenotype Cancer Res 1998 58 1839 1842 9581822

[B36] Atchley DP Albarracin CT Lopez A Valero V Amos CI Gonzalez-Angulo AM Clinical and pathologic characteristics of patients with BRCA-positive and BRCA-negative breast cancer J Clin Oncol 2008 26 4282 4288 10.1200/JCO.2008.16.6231 18779615 PMC6366335

[B37] Claus EB Risch N Thompson WD Autosomal dominant inheritance of early-onset breast cancer. Implications for risk prediction Cancer 1994 73 643 651 10.1002/1097-0142(19940201)73:3<643::AID-CNCR2820730323>3.0.CO;2-5 8299086

[B38] van Diest PJ Baak JP Matze-Cok P Wisse-Brekelmans EC van Galen CM Kurver PH Reproducibility of mitosis counting in 2,469 breast cancer specimens: results from the Multicenter Morphometric Mammary Carcinoma Project Hum Pathol 1992 23 603 607 10.1016/0046-8177(92)90313-R 1592381

[B39] van Diest PJ No consent should be needed for using leftover body material for scientific purposes. For BMJ 2002 325 648 651 10.1136/bmj.325.7365.648 12242180

[B40] Rosen PP Tumor emboli in intramammary lymphatics in breast carcinoma: pathologic criteria for diagnosis and clinical significance Pathol Annu 1983 18 Pt 2 215 232 6674861

[B41] Van Diest PJ No consent should be needed for using leftover body material for scientific purposes. For BMJ 2002 325 648 651 10.1136/bmj.325.7365.648 12242180

[B42] Lauria R Perrone F Carlomagno C De Laurentiis M Morabito A Gallo C The prognostic value of lymphatic and blood vessel invasion in operable breast cancer Cancer 1995 76 1772 1778 10.1002/1097-0142(19951115)76:10<1772::AID-CNCR2820761014>3.0.CO;2-O 8625046

[B43] Braun M Flucke U Debald M Walgenbach-Bruenagel G Walgenbach KJ Holler T Detection of lymphovascular invasion in early breast cancer by D2-40 (podoplanin): a clinically useful predictor for axillary lymph node metastases Breast Cancer Res Treat 2008 112 503 511 10.1007/s10549-007-9875-2 18165897

[B44] Lee AK DeLellis RA Silverman ML Heatley GJ Wolfe HJ Prognostic significance of peritumoral lymphatic and blood vessel invasion in node-negative carcinoma of the breast J Clin Oncol 1990 8 1457 1465 2202788 10.1200/JCO.1990.8.9.1457

[B45] Orbo A Stalsberg H Kunde D Topographic criteria in the diagnosis of tumor emboli in intramammary lymphatics Cancer 1990 66 972 977 10.1002/1097-0142(19900901)66:5<972::AID-CNCR2820660528>3.0.CO;2-O 2167149

[B46] Clemente CG Boracchi P Andreola S Del VM Veronesi P Rilke FO Peritumoral lymphatic invasion in patients with node-negative mammary duct carcinoma Cancer 1992 69 1396 1403 10.1002/1097-0142(19920315)69:6<1396::AID-CNCR2820690615>3.0.CO;2-I 1311623

[B47] Gilchrist KW Gould VE Hirschl S Imbriglia JE Patchefsky AS Penner DW Interobserver variation in the identification of breast carcinoma in intramammary lymphatics Hum Pathol 1982 13 170 172 10.1016/S0046-8177(82)80121-4 7076201

[B48] Birgisdottir V Stefansson OA Bodvarsdottir SK Hilmarsdottir H Jonasson JG Eyfjord JE Epigenetic silencing and deletion of the BRCA1 gene in sporadic breast cancer Breast Cancer Res 2006 8 R38 10.1186/bcr1522 16846527 PMC1779478

[B49] Catteau A Harris WH Xu CF Solomon E Methylation of the BRCA1 promoter region in sporadic breast and ovarian cancer: correlation with disease characteristics Oncogene 1999 18 1957 1965 10.1038/sj.onc.1202509 10208417

[B50] Esteller M Silva JM Dominguez G Bonilla F Matias-Guiu X Lerma E Promoter hypermethylation and BRCA1 inactivation in sporadic breast and ovarian tumors J Natl Cancer Inst 2000 92 564 569 10.1093/jnci/92.7.564 10749912

[B51] Matros E Wang ZC Lodeiro G Miron A Iglehart JD Richardson AL BRCA1 promoter methylation in sporadic breast tumors: relationship to gene expression profiles Breast Cancer Res Treat 2005 91 179 186 10.1007/s10549-004-7603-8 15868446

[B52] Bianco T Chenevix-Trench G Walsh DC Cooper JE Dobrovic A Tumour-specific distribution of BRCA1 promoter region methylation supports a pathogenetic role in breast and ovarian cancer Carcinogenesis 2000 21 147 151 10.1093/carcin/21.2.147 10657950

[B53] Snell C Krypuy M Wong EM Loughrey MB Dobrovic A BRCA1 promoter methylation in peripheral blood DNA of mutation negative familial breast cancer patients with a BRCA1 tumour phenotype Breast Cancer Res 2008 10 R12 10.1186/bcr1858 18269736 PMC2374968

[B54] Honrado E Benitez J Palacios J The molecular pathology of hereditary breast cancer: genetic testing and therapeutic implications Mod Pathol 2005 18 1305 1320 10.1038/modpathol.3800453 15933754

[B55] Prati R Apple SK He J Gornbein JA Chang HR Histopathologic characteristics predicting HER-2/neu amplification in breast cancer Breast J 2005 11 433 439 10.1111/j.1075-122X.2005.00125.x 16297088

